# Life-course trajectories of weight and their impact on the incidence of type 2 diabetes

**DOI:** 10.1038/s41598-021-91910-z

**Published:** 2021-06-14

**Authors:** Diego Yacamán-Méndez, Ylva Trolle-Lagerros, Minhao Zhou, Antonio Monteiro Ponce de Leon, Hrafnhildur Gudjonsdottir, Per Tynelius, Anton Lager

**Affiliations:** 1 Department of Global Public Health, Karolinska Institutet, Stockholm, Sweden; 2 Centre for Epidemiology and Community Medicine, Region Stockholm, Stockholm, Sweden; 3 Clinical Epidemiology Unit, Department of Medicine Solna, Karolinska Institutet, Stockholm, Sweden; 4 Obesity Centre, Academic Specialist Centre, Stockholm Health Services, Stockholm, Sweden

**Keywords:** Diabetes, Obesity, Epidemiology

## Abstract

Although exposure to overweight and obesity at different ages is associated to a higher risk of type 2 diabetes, the effect of different patterns of exposure through life remains unclear. We aimed to characterize life-course trajectories of weight categories and estimate their impact on the incidence of type 2 diabetes. We categorized the weight of 7203 participants as lean, normal or overweight at five time-points from ages 7–55 using retrospective data. Participants were followed for an average of 19 years for the development of type 2 diabetes. We used latent class analysis to describe distinctive trajectories and estimated the risk ratio, absolute risk difference and population attributable fraction (PAF) associated to different trajectories using Poisson regression. We found five distinctive life-course trajectories. Using the stable-normal weight trajectory as reference, the stable overweight, lean increasing weight, overweight from early adulthood and overweight from late adulthood trajectories were associated to higher risk of type 2 diabetes. The estimated risk ratios and absolute risk differences were statistically significant for all trajectories, except for the risk ratio of the lean increasing trajectory group among men. Of the 981 incident cases of type 2 diabetes, 47.4% among women and 42.9% among men were attributable to exposure to any life-course trajectory different from stable normal weight. Most of the risk was attributable to trajectories including overweight or obesity at any point of life (36.8% of the cases among women and 36.7% among men). The overweight from early adulthood trajectory had the highest impact (PAF: 23.2% for woman and 28.5% for men). We described five distinctive life-course trajectories of weight that were associated to increased risk of type 2 diabetes over 19 years of follow-up. The variability of the effect of exposure to overweight and obesity on the risk of developing type 2 diabetes was largely explained by exposure to the different life-course trajectories of weight.

## Introduction

The prevalence of high body weight, including overweight and obesity, has increased considerably over the last several decades, making it one of the main contributors to the global burden of disease and a pressing public health concern^1, 2^. Although there is a strong association between high body weight and increased risk of type 2 diabetes, the mechanisms underlying this association remain unclear^3, 4^, and so does the understanding of differences in the risk of type 2 diabetes between individuals^5^.

Weight has an important variability through life and a person’s body weight is the result of a complex combination of social, behavioral and genetic factors^6, 7^. Therefore, the consequences of exposure to high body weight are also heterogeneous^8, 9^. Although overweight and obesity considerably increase the risk of type 2 diabetes, most overweight and obese individuals never develop this complication^5^. From a public health perspective, there is a need for more precise identification of people whit a higher risk of type 2 diabetes.

Epidemiological evidence of the association between body weight and type 2 diabetes usually comes from studies linking weight status at a certain point in time or some measure of cumulative exposure to the occurrence of type 2 diabetes, typically several years later in life^10–14^. The segmented examination of the effects of high body weight comes with important limitations. Studies looking into a single time of exposure ignore the time-varying effects of changes in weight through different stages of life, while measures of cumulative exposure assume a constant effect at different ages, ignoring important physiological changes in metabolism as well as in social and behavioral factors related to age.

The use of longitudinal data and analyses has partially addressed these limitations^15–17^. However, longitudinal studies usually ignore the important differences observed between individuals exposed to overweight or obesity by generalizing the effect of exposure across the population. Furthermore, studies covering the whole life span, from childhood to adulthood, are scarce.

Identifying characteristics patterns of changes in weight through life by grouping individuals into more homogeneous sub-groups can lead to better-targeted and more precise public health strategies^18, 19^. Previous studies have examined the existence of sub-populations of individuals based on the developmental trajectories of weight at different points of life^20–22^, and some have investigated the association between different trajectories and the occurrence of type 2 diabetes^23–26^. However, little is known about the public health impact of different life-course weight trajectories.

Therefore, we aimed to ascertain characteristics life-course trajectories of weight from childhood to adulthood, estimate their association to the risk of type 2 diabetes, and to evaluate their effect at a population level.

## Results

The study sample for this analysis included 7203 (4820 women and 2383 men) participants of the Stockholm Diabetes Prevention Program cohort (SDPP) who received questions about recalled weight at different ages. During the study period, 65 (0.9%) participants were lost to follow-up.

**Figure 1:**
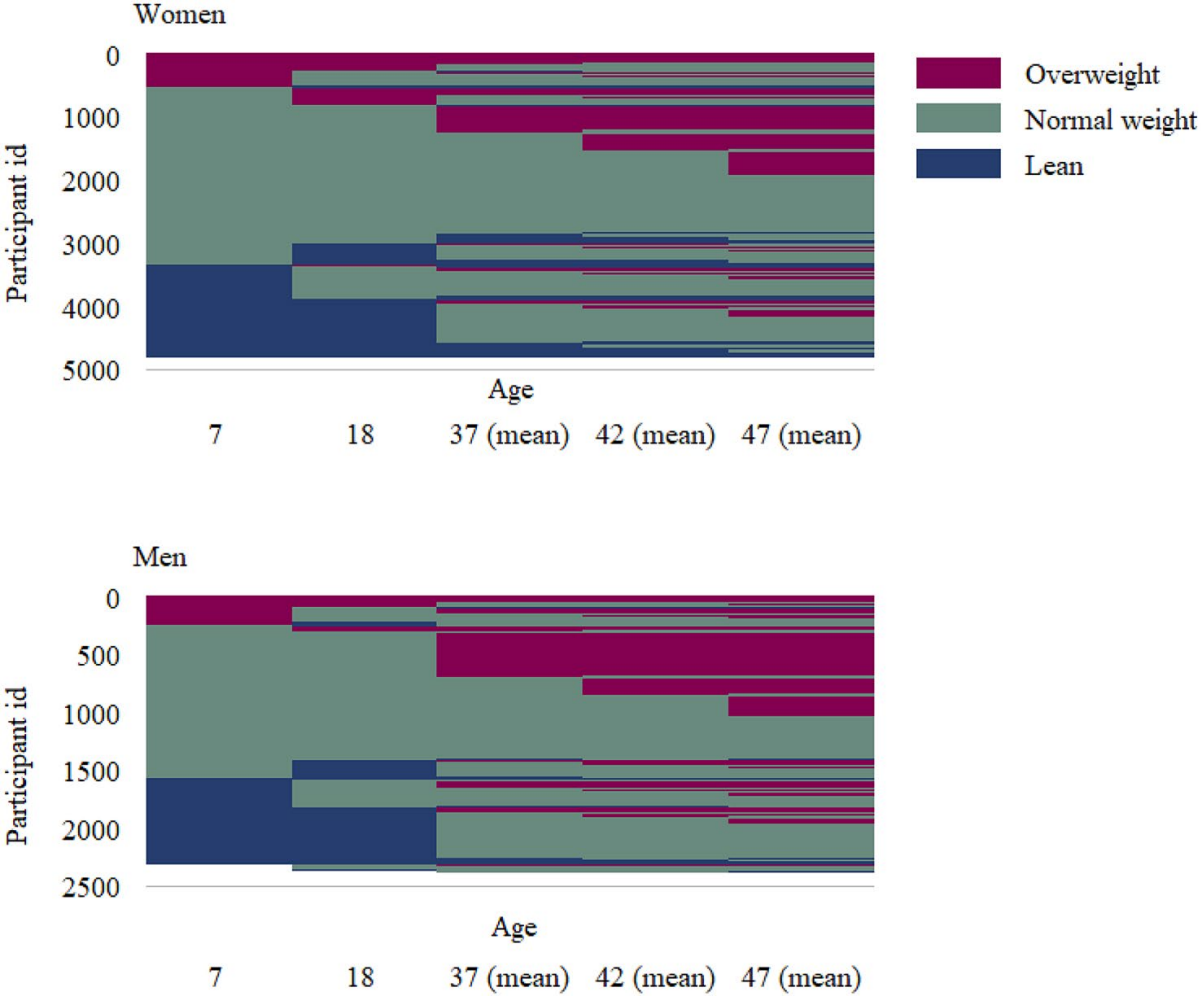
Individual weight categories at each of the study time-points. Categories of body weight were self-reported for childhood (7 years), adolescence (18 years), ten years before the study baseline (37 years in average) and five years before the study (42 years in average), and measured during the baseline examination (47 years in average). The individual participants are listed in the Y-axis; a change in color along time-points in the X-axis represents a change of category for an individual.

Self-reported categories of body weight at ages 7 and 18, body mass index (BMI) at ages 37 and 42, as well as measured BMI at a mean age 47 were used to ascertain the most common patterns of life-course development of weight categories. Figure 1 shows the individual level transitions between weight categories at each time point. A detailed description of recalled weight categories at each age is available online as supplemental Table S1.

**Figure 2:**
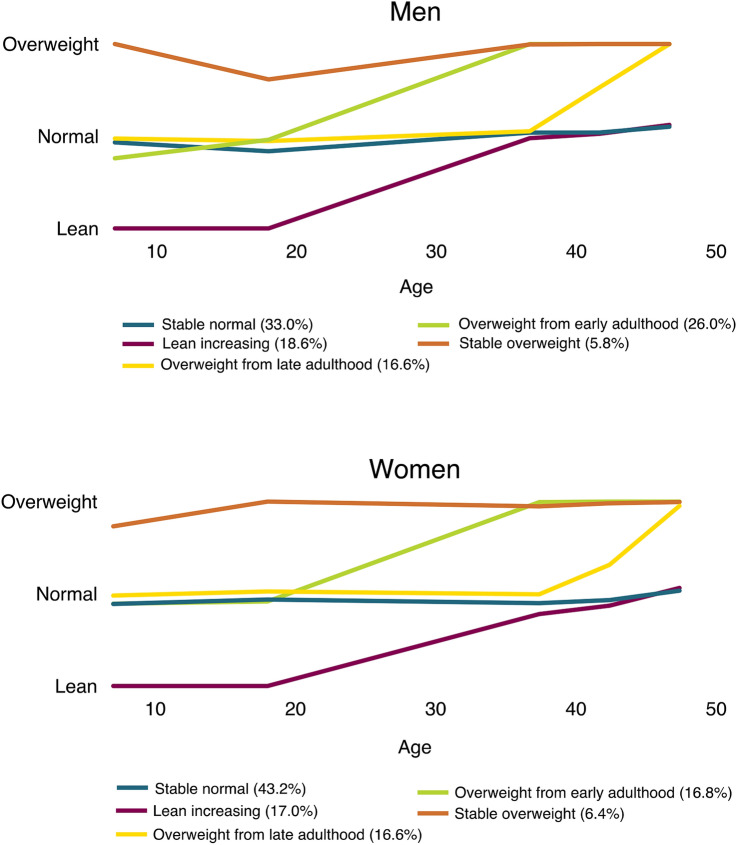
Life-course trajectories of weight categories by sex. The y-axis represents the probability of belonging to each weight category (lean, normal weight and overweight) estimated using group-based trajectory models. The predicted proportion of the population in each trajectory group is presented in parenthesis.

Using group-based trajectory modelling (GBTM), we found five distinct life-course weight trajectories, which exhibited similar patterns of changes for both sexes; although, there were differences in the proportion of the population within each trajectory group (Fig. 2).

The five trajectories were labelled based on their particular patterns as: (1) a stable normal weight trajectory, with 43.2% of all women and 33.0% of all men, largely made up of those with a normal weight throughout their life-course. (2) A stable overweight trajectory, with 6.4% of all women and 5.8% of all men, characterized by overweight or obesity from childhood and continues to adulthood. (3) A lean increasing weight trajectory, including 17.0% of all women and 18.6% of all men, that largely consisted of those who were lean in childhood and increased in weight in early adulthood without ever reporting overweight or obesity. (4) An overweight from early adulthood trajectory, with 16.8% of all women and 26.0% of men who developed overweight or obesity between their 20 s and 30 s. And (5) An overweight from late adulthood trajectory, with 16.6% of all women and 16.6% of men, characterized by people developing overweight or obesity after the age of 40.

Table 1 describes the baseline characteristics of the participants in each trajectory group. All the covariates were significantly different between trajectories. In comparison to the other trajectories, participants in the stable high weight and the high weight from early adulthood trajectory groups were characterized by having higher BMIs, higher systolic and diastolic blood pressure, lower levels of physical activity and educational attainment, and worse self-reported health at the time of the baseline examination for this study. Moreover, subjects in these groups had a higher prevalence of comorbidities, and a higher proportion had a family history of diabetes, as well as of gestational diabetes among women.

**Table 1:** Baseline characteristics of the study participants.

	Stable normal weight	Stable overweight	Lean increasing weight	Overweight from early adulthood	Overweight from late adulthood
	(n = 2966)	(n = 469)	(n = 1279)	(n = 1497)	(n = 992)
**Sex n (%)**					
*Female*	2158 (72.8%)	328 (69.9%)	833 (65.1%)	843 (56.3%)	658 (66.3%)
*Male*	808 (27.2%)	141 (30.1%)	446 (34.9%)	654 (43.7%)	334 (33.7%)
**Mean age (SD)**	47.3 (5.1)	46.4 (4.9)	47.3 (4.8)	46.4 (5.3)	47.82 (3.6)
**Mean BMI (SD)**	23.2 (1.9)	31.5 (4.9)	23.2 (2.5)	29.6 (3.4)	27.0 (1.9)
**Family history of diabetes n (%)**	1430 (48.2%)	299(63.8%)	658 (51.4%)	862 (57.6%)	558 (56.3%)
**History of gestational diabetes n (%)**	58 (2.7%)	20 (6.1%)	28 (3.4%)	66 (7.8%)	20 (3.0%)
**Self-reported general health n (%)**					
*Very good*	1358 (45.8%)	156 (33.3%)	518 (40.5%)	529 (35.4%)	372 (37.6%)
*Good*	1431 (48.3%)	264 (56.3%)	641 (50.2%)	803 (53.7%)	536 (54.1%)
*Neither good nor bad*	132 (4.5%)	37 (7.9%)	90 (7.0%)	122 (8.2%)	57 (5.8%)
*Bad or very bad*	44 (1.5%)	12 (2.6%)	29 (2.3%)	42 (2.8%)	25 (2.5%)
**Chronic disease n (%)**	709 (24.0%)	151 (32.3%)	387 (30.4%)	455 (30.7%)	290 (29.4%)
**Education level n (%)**					
*Primary or secondary education*	863 (29.1%)	175 (37.4%)	391 (30.7%)	584 (39.2%)	365 (36.9%)
*High school*	1065 (36.0%)	182 (38.9%)	460 (36.1%)	575 (38.6%)	370 (37.4%)
*University or higher*	1034 (34.9%)	111 (23.7%)	424 (33.3%)	331 (22.2%)	254 (25.7%)
**Mean systolic blood pressure (mmHg)**	118.8 (14.9)	128.0 (16.3)	120.9 (15.4)	127.6 (16.0)	125.0 (15.1)
**Mean diastolic blood pressure (mmHg)**	74.3 (9.4)	80.2 (10.1)	75.6 (9.7)	80.0 (10.0)	78.4 (9.3)
**Self-reported physical activity n (%)**					
*Much less*	100 (3.4%)	48 (10.3%)	54 (4.2%)	119 (8.0%)	63 (6.4%)
*Somewhat less*	428 (14.5%)	122 (26.1%)	264 (20.7%)	379 (25.4%)	198 (20.0%)
*Around the same*	1341 (45.3%)	202 (43.2%)	572 (44.8%)	667 (44.7%)	454 (45.8%)
*Somewhat more*	835 (28.2%)	84 (17.9%)	316 (24.7%)	264 (17.7%)	232 (23.4%)
*Much more*	257 (8.7%)	12 (2.6%)	72 (5.6%)	63 (4.2%)	44 (4.4%)
**Smoking category n (%)**					
*Never smoked*	1152 (38.9%)	176 (37.6%)	473 (37.0%)	540 (36.1%)	352 (35.5%)
*Current smoker*	1005 (33.9%)	164 (35.0%)	499 (39.0%)	568 (38.0%)	409 (41.2%)
*Previous smoker*	807 (27.2%)	128 (27.4%)	306 (23.9%)	388 (25.9%)	231 (23.3%)

### Incidence of type 2 diabetes

Over an average follow-up of 19 years for women and 21 years for men, a total of 981 incident cases of type 2 diabetes (536 among women and 445 among men) were identified by clinical examinations, linkage to regional inpatient and outpatient health-care registers or by self-report. Self-report was the only source of diagnosis information for 2.3% (23) of the recorded cases of type 2 diabetes.

The cumulative incidence of type 2 diabetes was 13.74% and was higher among men (18.7%) than women (11.3%). A detailed description of the cumulative incidence in each trajectory group is presented in Table 2.

**Table 2:** Cumulative incidence of type 2 diabetes by sex and life-course trajectories of weight.

	Total			Women			Men		
	Sample size	Cases	Cumulative incidence (%)	Sample size	Cases	Cumulative incidence (%)	Sample size	Cases	Cumulative incidence (%)
Stable normal weight	2952	196	6.64	2144	111	5.18	808	85	10.52
Stable overweight	454	104	22.91	313	62	19.81	141	42	29.78
Lean increasing weight	1270	143	11.26	824	84	10.19	446	59	13.23
Overweight from early adulthood	1476	383	25.95	822	184	22.38	654	199	30.42
Overweight from late adulthood	986	155	15.72	652	95	14.57	334	60	17.96
Total	7138	981	13.74	4755	536	11.3	2383	445	18.7

The results from the regression analyses showed that, relative to the stable normal weight trajectory group, the estimated risk ratio (RR) for diabetes under the study period was higher for all the trajectory groups. This trend was statistically significant for all trajectories except the lean increasing weight trajectory in men. While adjustments for confounders resulted in a modest decrease of the point estimates, they had no influence on the overall direction of the effect.

Estimates of RR were higher among women than men. The highest RR in the fully adjusted model were found in the overweight from early adulthood trajectory with an RR of 3.43 (95% CI 2.72–4.34) among women and 2.77 (95% CI 2.17–3.37) in men, and in the stable overweight trajectory with an RR of 2.77 (95% CI 2.06–3.72) for women and 2.68 (95% CI 1.92–3.75) for men.

In the fully adjusted model, the absolute risk difference (ARD) under the study period was significantly higher in all trajectories compared to the stable normal trajectory. The highest estimates were for the overweight from early adulthood trajectory, with 7.76% (95% CI 5.36–10.17%) more cases among women and 14.05% (95% CI 10.30–17.79%) among men. Followed by the stable overweight trajectory with an estimated ARD of 7.94% (95% CI 4.65–11.24%) more cases in women and 13.11% (95% CI 6.23–19.99%) in men. All the results from the adjusted and unadjusted modified Poisson regression models are summarized in Table 3. The ARD of a given trajectory in comparison to the stable normal trajectory was, in contrast to the RR, higher in men than women. This indicates that there was a larger effect of the weight trajectories among women, but an overall higher incidence of type 2 diabetes among men.

**Table 3:** Association between life-course trajectories of weight categories and cumulative incidence of type 2 diabetes.

Risk ratios (RR)	Women		Men	
	Unadjusted RR (95% CI)	Adjusted RR (95% CI)	Unadjusted RR (95% CI)	Adjusted RR (95% CI)
Stable normal weight	1 (ref)	1 (ref)	1 (ref)	1 (ref)
Stable overweight	3.69 (2.76–4.95)	2.77 (2.06–3.72)	2.94 (2.12–4.08)	2.68 (1.92–3.75)
Lean increasing weight	1.98 (1.50–2.60)	1.71 (1.31–2.24)	1.32 (0.96–1.81)	1.35 (0.98–1.85)
Overweight from early adulthood	4.07 (3.25–5.11)	3.43 (2.72–4.34)	3.03 (2.40–3.84)	2.77 (2.17–3.37)
Overweight from late adulthood	2.78 (2.13–3.63)	2.27 (1.75–2.95)	1.76 (1.29–2.40)	1.73 (1.26–2.37)

Absolute risk difference (ARD)	Unadjusted ARD% (95% CI)	Adjusted ARD% (95% CI)	Unadjusted ARD% (95% CI)	Adjusted ARD% (95% CI)
Stable normal weight	0 (ref)	0 (ref)	0 (ref)	0 (ref)
Stable overweight	10.90% (6.97%-14.83%)	7.94% (4.65%-11.24%)	15.05% (7.89%-22.21%)	13.11% (6.23%-19.99%)
Lean increasing weight	3.35% (1.34%-5.27%)	2.46% (0.57%-4.36%)	3.92% (0.05%-7.36%)	4.61% (0.95%-8.28%)
Overweight from early adulthood	9.03% (6.63%-11.40%)	7.76% (5.36%-10.17%)	15.23% (11.52%-18.95%)	14.05% (10.30%-17.79%)
Overweight from late adulthood	5.02% (2.66%-7.38%)	3.512% (1.36%-5.68%	4.84% (0.85%-8.82%)	5.23% (1.03%-9.43%)

Models for males and females were estimated separately. Covariates included in the adjusted model for were age, family history of type 2 diabetes, general health, comorbidities, self-reported physical activity, smoking, and alcohol consumption for both sexes and history of gestational diabetes for women. Adjusted absolute risk differences were estimated using the mean of all covariates.

### Population attributable fraction (PAF)

Overall, nearly half of the cases of diabetes among women (PAF: 47.40%, 95% CI 38.06%–55.34%) and men (PAF: 42.91%, 95% CI 31.47%–52.45%) were attributed to exposure to any life-course trajectory other than the stable normal. The trajectories including exposure to overweight or obesity at any point of life were associated to the highest population attributable fraction in both sexes (PAF: 36.82%, 95% CI 29.48%–43.40% for women and PAF: 36.71%, 95% CI 28.08%–44.31% for men).

**Figure 3:**
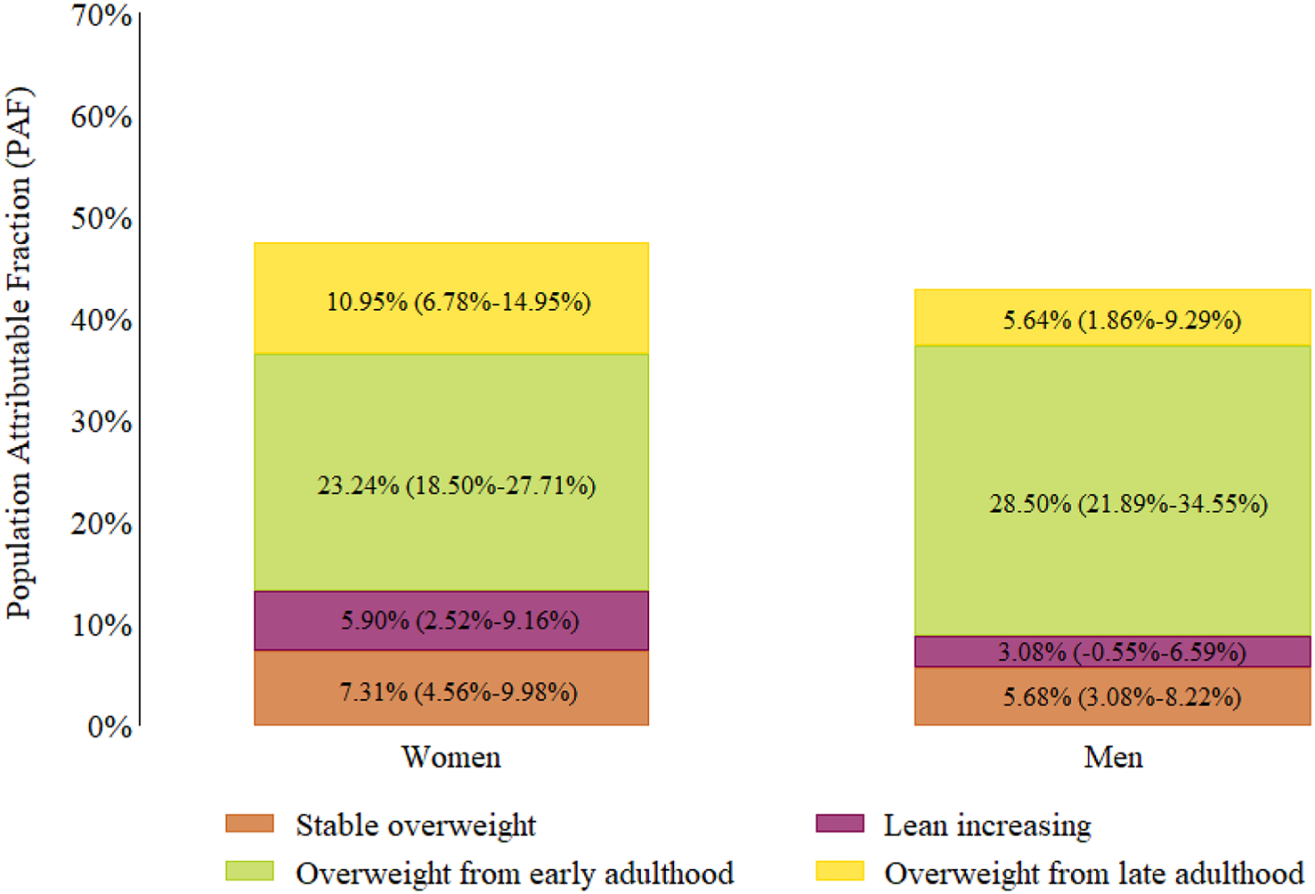
Population attributable fractions (PAF) of trajectory groups by sex. Compared to the stable normal trajectory group, the overall proportion of type 2 diabetes cases attributable to any of the life-course weight trajectories was 47.40% (95% CI 38.06–55.34%) for women and 42.91% (95% CI 31.47–52.45%) for men.

Individually, the PAF was 7.31% (95% CI 4.56%–9.98%) for the stable overweight trajectory, 5.90% (95% CI 2.52%–9.16%) for the lean increasing weight trajectory, 23.24% (95% CI 18.50%–27.71%) for the overweight from early adulthood group, and 10.95% (95% CI 6.78%–14.95%) for the overweight from late adulthood trajectory group among women. In men, the PAF was 5.68% (95% CI 3.08%–8.22%) for the stable overweight trajectory, 3.08% (95% CI − 0.55%–6.59%) for the lean increasing weight trajectory, 28.50% (95% CI 21.89%–34.55%) for the overweight from early adulthood trajectory, and 5.64% (95% CI 1.86%–9.29%) for the overweight from late adulthood trajectory group (Fig. 3).

## Discussion

We identified five distinct life-course trajectories of weight categories using data from a large cohort spanning from ages 7–55. We then determine the association and public health impact of these trajectories with prospective risk of type 2 diabetes.

We found that the variability of the effect of exposure to overweight and obesity on the risk of developing type 2 diabetes can be largely explained by the different life-course trajectories of exposure. Around half of the cases of type 2 diabetes were attributed to exposure to any life-course trajectory other than a stable normal weight though life. The trajectories including overweight or obesity at any point through the life-course accounted for most of the attributable proportion of cases in the population.

Childhood overweight continued to adulthood was associated to a high risk of type 2 diabetes among those exposed, but a low population attributable proportion. While the trajectory of overweight or obesity developed in early adulthood was associated to both a high risk of type 2 diabetes and population attributable proportion.

We found a higher estimated risk of type 2 diabetes, although not significant among men, for the lean increasing trajectory, despite never reporting overweight or obesity. The trajectory developing overweight or obesity later in adulthood was also associated with an increased risk of type 2 diabetes, but had a rather low impact at the population level.

### Strengths and limitations

The SDPP cohort study has compiled extensive clinical, self-reported and register data for a large, community-representative sample of healthy adults followed over 20 years for the development of type 2 diabetes. The use of inpatient and outpatient registries allowed us to obtain information regarding a new diagnosis of type 2 diabetes, even among participants who dropped out of the study. Loss to follow-up, an important source of bias in longitudinal studies was therefore minimized.

Another related strength was the ability to accurately estimate the incidence of type 2 diabetes. Ascertaining incidence is challenging due the long and insidious initial manifestations of the disease, and under diagnosis is common. In the SDPP, we used a combination of repeated OGTTs (the gold standard for the diagnosis of type 2 diabetes), individual level data linked to inpatient and outpatient registries, and self-reports of new diagnoses. This reduced the possibility of misclassifying the outcome, or over- or underestimating the estimated effects.

One limitation of the study is the fact that SDPP is a closed cohort and includes only participants born between 1938 and 1961. The prevalence of childhood obesity has increased importantly since then, which might affect the external generalizability of this study. Another important limitation of our study concerns the retrospective collection of data for childhood and adolescent weight categories, as well as weight 10 and 5 years previous to the study’s baseline examination. This information was, therefore, subject to recall bias. This could have led to differential misclassification of the exposure, which might have been more important among the males, who answered the question regarding childhood and adolescence weight during the 10-year follow-up and, at this stage, some of them had already developed type 2 diabetes. The women, however, answered questions regarding recalled weight at baseline, before any diagnosis of type 2 diabetes.

To use data from the entire life span, the data from recalled weight status from the baseline questionnaire, as well as recalled and measured BMIs, were categorized as lean, normal weight or overweight. This may have been an oversimplification and, therefore, it is possible that some degree of misclassification of the exposure was present in each category. Nonetheless, the prevalence of overweight and obesity during childhood reported by our participants was similar to that previously described in another Swedish study^27^.

There are also some limitations related to the use of GBTM^28^. First, although the variables used to categorize weight were ordinal, they were handled as censored normal in the statistical models. This approach has been described as reliable when dealing with longitudinal, categorical data^29^. In addition, classification in a certain trajectory group could have been conditional on age, as body weight normally increases with age, which would potentially explain why the higher risk groups were older at baseline. We addressed this limitation by adjusting for age at baseline. Another common issue with models based on finite mixture models is that exposure is not directly observed, but rather individuals are assigned to the different groups based on their posterior class probabilities according to the selected model. This might create bias due to misclassification of the exposure. However, the different methods used to relate latent classes to auxiliary variable, such as the three-step method used in this study, have been shown to underestimate the observed associations, which would in any case lead to a type 2 error^30^.

Finally, as with all generalizations, there is a trade-off between complexity and interpretability of the data. The trajectories presented in this study are a useful generalization of a complex underlying longitudinal process and attempt to describe the most characteristic patterns at the population level. At an individual level, however, several different trajectories exist. Individual level patterns within each trajectory group of a sub-sample of the participants is available online as supplemental material as Figure S1. Overall, when using these methods, it is important to be cautious in the interpretation of the trajectory groups, since the external generalizability might be limited. Comparison with previous studies is therefore important^28^.

### Comparison with previous literature

The life-course trajectories described in this study are consistent with those previously reported in the literature. Several studies have used repeated measures of BMI, waist circumference or body shape to approximate unobserved sub-groups of developmental trajectories of weight through different periods of life and estimated their association to different outcomes, including type 2 diabetes^31^.

Among the studies looking into the association between the underlying trajectories of weight and risk of type 2 diabetes. One study used prospective BMI data from a large cohort of white civil servants from Britain and found three distinctive trajectories associated to a higher risk of type 2 diabetes^23^. Another study using retrospective BMI data from a Chinese population of adults followed for 6 years found 4 distinctive trajectories associated to higher risk of type 2 diabetes^32^. And a study that used retrospective data from a cohort of Australian adult women reported three trajectories of BMI that were associated to the risk of type 2 diabetes^33^. However, these studies did not include information from body weight during childhood or adolescence, ignoring the life-course effects of different patterns of changes in weight or body composition.

Studies covering larger periods of life also exist. A study using retrospective data from two large cohorts in the United States reported five trajectories of body shape from ages 5–55 associated to increased risk of type 2 diabetes^24^. Another similar study used data from a large cohort of women in France reporting five trajectories of self-reported body shapes from ages 8–40 associated to higher risk of type 2 diabetes^26^. And a study using prospective BMI data from Finland from ages ranging from 6 to 49 years reported six trajectories and their association to the risk of type 2 diabetes and other cardiovascular outcomes^21^. However, these studied estimated only relative measures of effect. Our study adds to the literature by estimating absolute measures of association (absolute risk difference and population attributable fraction), which provide information regarding the potential public health impact of the exposure to different patterns of weight categories through life.

The different life-course trajectories of exposure helped explained the variability of the effect of exposure to overweight and obesity on the risk of developing type 2 diabetes. Weight gains at different ages had a significant association to the risk and public health impact on the incidence of type 2 diabetes. The highest risk and public health impact was among those who became overweight or obese during early adulthood. Although previous studies have pointed out the importance of high body weight during early adulthood and the risk of type 2 diabetes^13, 21^, the public health impact of this period is often neglected. Our findings point out to early adulthood as an important period for public health interventions to reduce the burden of type 2 diabetes related to high body weight.

The stable overweight trajectory group (i.e. to overweight or obesity from childhood continued to adulthood) was associated to a high risk of type 2 diabetes among those exposed, but to a low public health impact in the population. These findings are consistent with existing literature, a recent meta-analysis of 37 studies concluded that, while there was a moderate association between childhood obesity and type 2 diabetes in adulthood, the public health relevance is limited^10^.

Interestingly, we found an increased risk, although not significant for men, among people who were not overweight or obese, but increased from lean to normal weight during their early adulthood. Other studies have previously pointed out the importance of weight changes on the risk of type 2 diabetes even among lean individuals^34^.

### Implications for future research and public health

Our findings suggests at least two different underlying mechanisms of the effect of overweight on type 2 diabetes. A cumulative effect from childhood to adulthood, and a sensitive period, early adulthood, when exposure to changes in weight status, even among non-overweight or obese individuals, seems to have a greater effect.

We provide further evidence for the importance of a life-course approach in public health. Rather than investigating the effects of exposure at a single time point or stage of development, studying the effects of overweight and obesity as a continuous exposure during the life-course might help to better understand the natural history of its association to type 2 diabetes and point towards different causal mechanisms^18^.

Describing the determinants that differentiate these trajectories as well as their association to other health outcomes can aid in the design of more precise and targeted interventions to influence an individual´s health most efficiently^18^.

However, the categories presented in this study are not expected to be stable through generations. The prevalence of childhood obesity has increased in Sweden and around the world drastically during the past few decades^27, 35^. What is the effect of the increased prevalence of childhood obesity on the life-course trajectories of weight and on the incidence of type 2 diabetes of younger cohorts remains an important area for further research.

## Research design and methods

### Study population

Data for this study comes from the clinical subsample of the Stockholm Diabetes Prevention Program (SDPP), a 20-year prospective study that followed healthy individuals living in Stockholm, a detailed description of SDPP can be found elsewhere^36^.

In short, 16,481 healthy women and men from five selected municipalities in Stockholm, who were between 35 and 56 years old were identified between 1992 and 1998 using the National Population Registry of Sweden. A clinical subsample was created including 7948 participants, born in Sweden, with a family history of type 2 diabetes, women with a history of gestational diabetes, and a randomly selected sample of controls without a family history of type 2 diabetes.

Clinical examinations have been conducted at baseline, 10-years follow-up and 20-years follow-up and included extensive questionnaires, oral glucose tolerance test (OGTT), anthropometric and blood pressure measurements, and blood sample collection. Additionally, the personal identification number of all baseline participants was used to link individual level data to the regional data base of healthcare utilization, the *Vårdanalysdatabasen* (VAL) registry, which includes information about inpatient and outpatient services of Stockholm, including all diagnostics according to the International Classification of Diseases, tenth revision (ICD-10)^37^.

The sample for the present study includes 7203 participants (4820 women and 2383 men) who received the questionnaire including questions about their recalled weight status at ages 7 and 18. For women, this question was included in the baseline questionnaire while for men in the 10-year follow-up study.

### Variables

#### Weight categories through life

To estimate the life course trajectories of weight categories, we used self-reported weight status at ages 7 and 18, recalled weight 10 and 5 years prior to the baseline examination and measured BMI from the baseline examination. In order to allow the analysis using all time points, the data at each time point was categorized into lean, normal weight or overweight as specified below.

#### Self-Reported weight status during childhood and adolescence

Participants answered questions about their weight at ages 7 and 18 during the baseline examination for women and on the 10-year follow-up questionnaire for men. The specific ages were selected in order to represent childhood and the end of adolescence. The question was the same for both sexes: “Compared to others of your same age, what was your weight status at age 7 and 18”. The answer categories were given as a Likert scale with the values: (1) very lean, (2) somewhat lean, (3) normal weight, (4) somewhat overweight, (5) very overweight. Similar recall measurements of childhood weight status have previously been validated^38, 39^. We categorized very lean and somewhat lean as lean, normal weight was kept as a category, and somewhat overweight and very overweight were categorized as overweight for each age.

#### Self-Reported weight 10 and 5 years before baseline

During the baseline examination, participants were asked to report their recalled weight 10 and 5 years prior to study inclusion. BMI was then calculated as BMI = weight (kg)/height^2^ (m) using measured height from the baseline examination. BMI values were categorized as lean (BMI < 19.5), normal weight (BMI ≥ 19.5 and < 25), and overweight (BMI ≥ 25)^40, 41^.

#### BMI during the baseline clinical examination

Weight and height for all participants was measured during the baseline clinical examination by trained study staff. BMI was calculated using measured weights and heights at each time point and participants were categorized as described above.

#### Incidence of type 2 diabetes

Incidence of type 2 diabetes was determined from three different sources: OGTTs performed during the clinical examinations, a new diagnosis of type 2 diabetes (ICD-10 code E11) in the VAL registry at any time after the baseline examination, or a self-reported new diagnosis of type 2 diabetes given by a health care professional during any of the study’s follow-up periods.

For the OGTT, we defined type 2 diabetes as a fasting plasma glucose level higher than or equal to 7 mmol/L or a 2 h, post-load plasma glucose level higher than or equal to 11 mmol/L during the OGTT^42^. During the 10- and 20-year follow-ups, only participants without a self-reported diagnosis of type 2 diabetes were offered a new OGTT.

#### Covariates

At baseline, we assessed the following possible confounders: age, educational level, self-reported general health, chronic comorbidities, family history of type 2 diabetes, physical activity, smoking status, alcohol consumption, and, for women, a history of gestational diabetes.

Age was available from the total population register of Sweden. Educational level was self-reported, categorized as primary education, upper secondary education, or university education, and higher. Self-reported general health was evaluated with the question: “How is your general state of health “and included the categories: “very good”, “good”, “neither good or bad” and “bad or very bad”. The presence of any self-reported comorbidities in the baseline questionnaire was used as a dichotomous variable. A family history of diabetes was self-reported, defined as having one first-degree relative or two second-degree relatives with the disease and was dichotomized as present or not present. Information regarding gestational diabetes was also obtained from the baseline questionnaire administered to women and was used as a dichotomous measure. Level of physical activity was assessed with the question “Compared to other men/women your age, how do you consider your physical activity in your spare time?” and categorized as “much lower”, “somewhat lower”, “about the same”, “slightly higher” and “much higher”. Smoking was self-reported and categorized as never smoked, previous smoker and current smoker, and alcohol consumption was calculated in cl per week from self-reported standard units and frequencies.

### Statistical analysis

Descriptive statistics are presented as means and standard deviations (SDs) for continuous variables and as proportions for binary and categorical data.

We used Group Based Trajectory Modelling (GBTM) to ascertain the life course trajectories of the five weight categories using the Traj procedure^43^ in Stata 14^44^. GBTM is an application of finite mixture modelling that is useful to study the development of an outcome over time. The main assumption is that an observed population is composed of underlying distinctive sub-populations (or latent classes) of individuals. And that the composition and characteristics of such sub-groups can be estimated using parametric methods^45^.

The model selection for the GBTM was done in multiple steps to identify the best number and structure of the trajectory groups. Trajectories were estimated separately for men and women given their differences in lipid metabolism, development of adiposity, and the effect of high body weight between men and women^46^.

Determination of the goodness-of-fit and parsimony of the model was based on a low Bayesian information criterion, a large Bayes factor, an average posterior probability of assignment to a given trajectory group greater than 75%, and a size of more than 1% in each group^47, 48^. After the number of trajectory groups and parameters of the models were selected, participants were assigned to the trajectory group to which they were more likely to belong based on their individual weight categories at all the time points.

Missing data in the weight categories at any time point was managed using the full information maximum-likelihood method based on the available data points under the missing at random assumption, i.e., missing was not related to the outcome, but was related to other observed variables^45^. Further information on the model selection process can be found online as Supplemental Tables S2–S4.

We investigated the association between the different life-course trajectories of weight categories and incidence of type 2 diabetes using modified Poisson regression with robust error variance^49^. The study period for this analysis extended from the date of baseline examination to the date of the third study follow-up, or until January 1st 2018 for those participants whose outcome status was retrieved from regional inpatient or outpatient registries. The mean follow-up time was 19 years for women and 21 years for men. We reported estimated risk ratios, absolute risk differences and population attributable fractions based on the cumulative incidence during the whole study period.

The connection between the exposure to the estimated trajectory groups and incidence of type 2 diabetes was done using a three-step approach: First, the selected GBTM model was fitted using only the latent class indicator variables, i.e. the weight categories at each of the five time points. Next, individuals were assigned to the trajectory group they were most likely to belong based on the posterior probability of group membership. Lastly, the modified Poisson regression models were fitted using the inverse probability of class membership as weights to adjust for classification uncertainty^30, 50^.

Covariates included in the adjusted model included age at baseline, self-reported physical activity, family history of diabetes, comorbidities, self-reported general health status, educational level, smoking and alcohol use. For women, additional adjustment was done for history of gestational diabetes.

The burden of type 2 diabetes attributable to each trajectory group was calculated using the population attributable fractions (PAF), defined as the proportion of cases in the population that can be attributed to a certain exposure. In other words, the proportion of cases that would not have occurred had the exposure not been present.

We calculated the PAF for exposure to any trajectory compared to the stable normal weight trajectory group according to the general formula:

$$PAF = \mathop \sum \limits_{i = 0}^{n} p_{i} \left( {\frac{{RR_{i} - 1}}{{RR_{i} }}} \right)$$

where *p*_i_ represents the proportion of cases in each level of exposure (i.e. each trajectory group) and *RR*_*i*_ represents the adjusted RR of type 2 diabetes for each trajectory group compared to the stable normal group.

All statistical analysis was done using Stata 14^51^. The user written commands *traj*^43^ and *punaf*^52^ were used to estimate the GBTM and PAFs, respectively.

### Ethics approval

This study was carried out in accordance with the ethical principles outlined in the Declaration of Helsinki^53^. The SDPP study received approval by the regional ethics review board of Stockholm (2013/1982-31/2).

All participants of the SDPP study received verbal and written information about the study and provided informed consent at the time of recruitment and at each follow-up visit.

## Supplementary Information

Supplementary Information.

## Data Availability

The datasets generated and/or analysed during the current study are available from the corresponding authors upon reasonable requests. The Centre or Epidemiology and Community Medicine of Region Stockholm manages the data from the Stockholm Diabetes Prevention Program. Sensitive information is protected by the general data protection regulation (GDPR). The code used for data analysis of this manuscript is available from the corresponding author upon reasonable request.

## Abbreviations

BMI: Body mass index
OGTT: Oral glucose tolerance test
ICD-10: The International Classification of Diseases, tenth revision
CI: Confidence interval
ARD: Absolute risk difference
RR: Risk ratio
GBTM: Group based trajectory modelling
PAF: Population attributable fraction
SDPP: Stockholm diabetes prevention program
VAL: Stockholm County central regional data base of healthcare utilization (Vårdanalysdatabasen)
