# Author Correction: Life-course trajectories of weight and their impact on the incidence of type 2 diabetes

**DOI:** 10.1038/s41598-021-98091-9

**Published:** 2021-09-14

**Authors:** Diego Yacamán-Méndez, Ylva Trolle-Lagerros, Minhao Zhou, Antonio Monteiro Ponce de Leon, Hrafnhildur Gudjonsdottir, Per Tynelius, Anton Lager

**Affiliations:** 1 Department of Global Public Health, Karolinska Institutet, Stockholm, Sweden; 2 Centre for Epidemiology and Community Medicine, Region Stockholm, Stockholm, Sweden; 3 Clinical Epidemiology Unit, Department of Medicine Solna, Karolinska Institutet, Stockholm, Sweden; 4 Obesity Centre, Academic Specialist Centre, Stockholm Health Services, Stockholm, Sweden

Correction to: *Scientific Reports* https://doi.org/10.1038/s41598-021-91910-z , published online 14 June 2021

The original version of this Article contained errors.

In the Results section, under the subheading ‘Population attributable fraction (PAF)’,

“The trajectories including exposure to overweight or obesity at any point of life were associated to the highest population attributable fraction in both sexes (PAF: 36.82%, 95% CI 0.29%–0.43% for women and PAF: 36.71%, 95% CI 28.08%–44.31% for men).”

now reads:

“The trajectories including exposure to overweight or obesity at any point of life were associated to the highest population attributable fraction in both sexes (PAF: 36.82%, 95% CI 29.48%–43.40% for women and PAF: 36.71%, 95% CI 28.08%–44.31% for men).”

In Table 2, the Total, Cumulative incidence (%) for “Stable normal weight” was incorrect.

“0.66”

now reads:

“6.64”

In Table 3, the values for Women, Adjusted RR (95% CI) for “Stable overweight” and “Overweight from early adulthood” and Men, Adjusted RR (95% CI) for “Overweight from early adulthood” were incorrect. Furthermore the value for Men, Adjusted ARD% (95% CI) for “Stable overweight” was incorrect. The original Table 3 and accompanying legend appear below.

In the legend of Figure 3,

“Compared to the stable normal trajectory group, the overall proportion of type 2 diabetes cases attributable to any trajectory of overweight or obesity through the life course was 47.40% (95% CI 38.06–55.34%) for women and 42.91% (95% CI 31.47–52.45%) for men.”

now reads:

“Compared to the stable normal trajectory group, the overall proportion of type 2 diabetes cases attributable to any of the life-course weight trajectories was 47.40% (95% CI 38.06–55.34%) for women and 42.91% (95% CI 31.47–52.45%) for men.”

Lastly, the Supplementary Table S2, S3 and S4 legends were omitted. The original Supplementary Information file is provided below.

The original Article and accompanying Supplementary Information file have been corrected.

**Table 3:** Association between life-course trajectories of weight categories and cumulative incidence of type 2 diabetes.

Risk ratios (RR)	Women		Men	
	Unadjusted RR (95%CI)	Adjusted RR (95%CI)	Unadjusted RR (95%CI)	Adjusted RR (95%CI)
Stable normal weight	1 (ref)	1 (ref)	1 (ref)	1 (ref)
Stable overweight	3.69 (2.76–4.95)	2.77 (2.06–3.73)	2.94 (2.12–4.08)	2.68 (1.92–3.75)
Lean increasing weight	1.98 (1.50–2.60)	1.71 (1.31–2.24)	1.32 (0.96–1.81)	1.35 (0.98–1.85)
Overweight from early adulthood	4.07 (3.25–5.11)	3.43 (2.72–3.34)	3.03 (2.40–3.84)	2.77 (2.17–3.54)
Overweight from late adulthood	2.78 (2.13–3.63)	2.27 (1.75–2.95)	1.76 (1.29–2.40)	1.73 (1.26–2.37)

Absolute risk difference (ARD)	Unadjusted ARD% (95%CI)	Adjusted ARD% (95%CI)	Unadjusted ARD% (95%CI)	Adjusted ARD% (95%CI)
Stable normal weight	0 (ref)	0 (ref)	0 (ref)	0 (ref)
Stable overweight	10.90% (6.97%–14.83%)	7.94% (4.65%–11.24%)	15.05% (7.89%–22.21%)	13.11% (6.24%–19.99%)
Lean increasing weight	3.35% (1.34%–5.27%)	2.46% (0.57%–4.36%)	3.92% (0.05%–7.36%)	4.61% (0.95%–8.28%)
Overweight from early adulthood	9.03% (6.63%–11.40%)	7.76% (5.36%–10.17%)	15.23% (11.52%–18.95%)	14.05% (10.30%–17.79%)
Overweight from late adulthood	5.02% (2.66%–7.38%)	3.512% (1.36%–5.68%)	4.84% (0.85%–8.82%)	5.23% (1.03%–9.43%)

Models for males and females were estimated separately. Covariates included in the adjusted model for were age, family history of type 2 diabetes, general health, comorbidities, self-reported physical activity, smoking, and alcohol consumption for both sexes and history of gestational diabetes for women. Adjusted absolute risk differences were estimated using the mean of all covariates.

## Supplementary Information

Supplementary Information.

