# Supplementary material for: Life-course trajectories of weight and their impact on the incidence of type 2 diabetes
Source: Sci Rep. 2021 Jun 14;11:12494. doi: 10.1038/s41598-021-91910-z (PMC8203741; doi:10.1038/s41598-021-91910-z)
Supplement: Supplementary file 1 — Supplementary Information. [file 41598_2021_91910_MOESM1_ESM.docx]

**Life-course trajectories of weight and their impact on the incidence of type 2 diabetes**

Diego Yacamán-Méndez*^1,2^, Ylva Trolle-Lagerros^3,4^, Minhao Zhou^2^, Antonio Monteiro Ponce de Leon^2^, Hrafnhildur Gudjonsdottir^2^, Per Tynelius^1,2^, Anton Lager^1,2^

^1^ Department of Global Public Health, Karolinska Institutet, Stockholm, Sweden.

^2^ Centre for Epidemiology and Community Medicine, Region Stockholm, Stockholm Sweden.

^3^ Clinical Epidemiology Unit, Department of Medicine Solna, Karolinska Institutet, Stockholm Sweden.

^4^ Obesity Centre, Academic Specialist Centre, Stockholm Health Services. Stockholm, Sweden.

ORCID identifiers:

Diego Yacamán-Méndez: 0000-0002-8130-0229

Ylva Trolle-Lagerros: 0000-0001-6816-7577

Hrafnhildur Gudjonsdottir: 0000-0001-8235-7238

Minhao Zhou: 0000-0001-8801-2847

Antonio Monteiro Ponce de Leon: 0000-0002-0704-5215

Per Tynelius: 0000-0001-8155-7406

Anton Lager: 0000-0001-5263-8063

Correspondence to:

Diego Yacamán-Méndez, Department of Global Public Health, Karolinska Institutet, Stockholm, Sweden. SE-171 77; Email: diego.yacaman.mendez@ki.se

**Supplemental material**

## Supplemental Table S1: Distribution of weight categories among women and men.

|  | **All participants** | **Women** | **Men** | **p-value** |
| --- | --- | --- | --- | --- |
| **Total study sample** | **n=7,203** | **n=4,820** | **n=2,383** |  |
| **7 years old** | **n=7,135** | **n=4,813** | **n=2,322** |  |
| Lean (%) | 2,198 (30.8%) | 1,463 (30.4%) | 735 (31.7%) | 0.45 |
| Normal weight (%) | 4,125 (57.8%) | 2,791 (58.0%) | 1,334 (57.5%) |  |
| Overweight (%) | 812 (11.4%) | 559 (11.6%) | 253 (10.9%) |  |
| **18 years old** | **n=7,188** | **n=4,815** | **n=2,373** |  |
| Lean (%) | 1,939 (27.0%) | 1,255 (26.1%) | 684 (28.8%) | <0.01 |
| Normal weight (%) | 4,466 (62.1%) | 2,946 (61.2%) | 1,520 (64.1%) |  |
| Overweight (%) | 783 (10.9%) | 614 (12.8%) | 169 (7.1%) |  |
| **10 years before baseline** | **n=7,124** | **n=4,763** | **n=2,361** |  |
| Mean Age (SD) | 37.13 (4.91) | 37.37 (4.91) | 36.64 (4.86) | <0.01 |
| Mean BMI (SD) | 23.34 (3.39) | 22.94 (3.52) | 24.16 (2.94) | <0.01 |
| Lean (%) | 554 (7.8%) | 494 (10.4%) | 60 (2.5%) | <0.01 |
| Normal weight (%) | 4,828 (67.8%) | 3,278 (68.8%) | 1,550 (65.7%) |  |
| Overweight (%) | 1,742 (24.5%) | 991 (20.8%) | 751 (31.8%) |  |
| **5 years before baseline** | **n=7,142** | **n=4,770** | **n=2,372** |  |
| Mean Age (SD) | 42.13 (4.91) | 42.37 (4.91) | 41.65 (4.86) | <0.01 |
| Mean BMI (SD) | 24.29 (3.67) | 23.99 (3.86) | 24.88 (3.16) | <0.01 |
| Lean (%) | 330 (4.6%) | 290 (6.1%) | 40 (1.7%) | <0.01 |
| Normal weight (%) | 4,331 (60.6%) | 3,010 (63.1%) | 1,321 (55.7%) |  |
| Overweight (%) | 2,481 (34.7%) | 1,470 (30.8%) | 1,011 (42.6%) |  |
| **Baseline** | **n=7,180** | **n=4,803** | **n=2,377** |  |
| Mean Age (SD) | 47.13 (4.91) | 47.37 (4.91) | 46.65 (4.86) | <0.01 |
| Mean BMI (SD) | 25.59 (4.04) | 25.43 (4.30) | 25.91 (3.42) | <0.01 |
| Lean (%) | 153 (2.1%) | 132 (2.7%) | 21 (0.9%) | <0.01 |
| Normal weight (%) | 3,429 (47.8%) | 2,445 (50.9%) | 984 (41.4%) |  |
| Overweight (%) | 3,598 (50.1%) | 2,226 (46.3%) | 1,372 (57.7%) |  |

For continuous values, mean and standard deviation in parenthesis are provided. For categorical variables, the total number and percentage in parenthesis are presented. Comparisons were made with t-test for continuous variables and Chi^2^ for categorical values. All p-values were calculated as two sided with a significant level of 0.05.

## Model selection for the GBTM

We used group-based trajectory modelling (GBTM) to identify life-course trajectories of weight status. GBTM is an application of finite mixture modelling to study the development of an outcome over time. The main assumption is that an observed population is actually composed of an underlying finite number of unknown distinctive sub-populations (or latent classes) of individuals ^1^.

One of the key analytical considerations when using finite mixture models, is the selection of the number of categories (or latent classes) that better describe the data. In this study, we used a multiple step model selection method ^2^.

First, we fitted models for 1 to 6 groups using a linear structure in order to determine the optimal number of trajectories. Once the number of trajectories was determined, we proceeded to identify the best polynomial structure by fitting linear, quadratic, cubic and quartic polynomials for the selected number of trajectories. Finally, we fitted all combinations of the two best polynomial structures to determine the best combination of polynomials.

At all stages, selection was based in the goodness of fit of the model, using the Bayesian Information Criterion (BIC) and Akaike Information Criterion (AIK). As well as the parsimony and interpretability of the model assessed with the approximated Bayes factor, defined as 2log(B_10_)≈2(ΔBIC)^3^, a posterior probability of assignment (OCC) greater than 75%, and by limiting the size to more than 1% of the population in each group ^4^. Supplemental tables 2 through 4 provide a detailed description of the model selection process.

The resulting trajectories are useful to display patterns of longitudinal data. However, they are a summary of an underlying complex process. The increased interpretability is obtained at the expense of grouping together individuals that are not entirely homogeneous^5^. Supplementary figure S2 provides a visual representation of the individual level variations in each of the trajectory groups by displaying the trajectories of a random sample of 25% of the population.

### Supplemental Table S2: Model selection step 1. Determining the number of trajectories (k)

| **Model** | **K** | **Structure** | **BIC** | **AIC** | **Bayes factor**  **2log(B10)** | **Predicted proportion per class** | **Posterior probability of class membership** |
| --- | --- | --- | --- | --- | --- | --- | --- |
| **Women** |  |  |  |  |  |  |  |
|  | 2 | linear | -26387.4 | -26368.0 | - | 73.0%, 27.0% | 97.5%, 91.6% |
|  | 3 | linear | -25979.0 | -25049.9 | 2611.9 | 18%, 56.6%, 25.5% | 94.7%, 95.6%, 92.0% |
|  | 4 | linear | -24660.6 | -24621.7 | 832.1 | 17.8%, 24.4%, 5.4%, 3.4% | 95.9%, 90.8%, 96.2%, 98.6% |
|  | **5** | **linear** | **-24208.6** | **-24145.0** | **899.11** | **16.7%, 22.3%, 3.42%, 53.3%, 4.3%** | **95.5%, 90.1%, 87.6%, 95.7%, 96.9%** |
|  | 6^*^ | linear | -24133.5 | -24075.2 | 145.3 | 12.6%, 6.1%, 22.6%, 52.6%, 2.7%, 3.4% | 92.2%, 84.8%, 91.9%, 97.0%, 90.1%, 98.2% |
|  | 7^*^ | linear | -24064.1 | -23996.0 | 134.1 | 16.4%, 3.4%, 22.6%, 44.0%, 2.7%, 3.4% | 95.1%, 88.3%, 89.8%, 82.5%, 90.9%, x%, 98.4% |
| **Men** | **K** | **Structure** | **BIC** | **AIC** | **Bayes factor**  **2log(B10)** | **Predicted proportion per class** | **Posterior probability of class membership** |
|  | 2 | linear | -11928.1 | -11910.8 | NA | 61.6%, 38.4% | 97.2%, 95.2% |
|  | 3 | linear | -11300.6 | -11274.6 | 1250.1 | 19.8%, 36.5%, 43.6% | 95.2%, 95.0%, 92.7% |
|  | 4 | linear | -11111.9 | -11077.2 | 372.7 | 19.8%, 34.5%, 42.6%, 3.1% | 95.0%, 94.7%, 93.2%, 94.9% |
|  | **5** | **linear** | **-10911.1** | **-10867.7** | **396.9** | **17.4%, 33.2%, 3.5%, 43.1%, 2.8%** | **97.4%, 95.1%, 92.0%, 93.4%, 97.1%** |
|  | 6 | linear | -10890.2 | -10838.2 | 36.9 | 17.9%, 3.6%, 7.8%, 28.3%, 39.7%, 2.7% | 96.1%, 91.2%, **68.4%,** 91.6%, 90.8%, 98.5% |
|  | 7 | linear | -10838.2 | -10777.5 | 99 | 3.6%, 17.4%, 7.8%, 39.1%, 28.0%, 1.5%, 2.8% | 92.8%, 97.2%, **70.9%,** 88.9%, 92.6%, 90.5%, 97.3% |

K denotes the number of trajectory groups, asterisk (*) = model failed to converge, BIC=Bayesian information criterion. AIC=Akaike information criterion.

### Supplemental Table S3: Model selection step 2. Gross determination of the structure of the k trajectories

| **Model** | **structure** | **BIC** | **AIC** | **Bayes factor**  **2log_e_(B10)** | **Predicted proportion per class** | **Posterior probability of class membership** |
| --- | --- | --- | --- | --- | --- | --- |
| **Women** |  |  |  |  |  |  |
|  | Linear | -24208.6 | -24160.0 | - | 16.7%, 22.3%, 3.4%, 53.3%, 4.3% | 95.5%, 90.1%, 87.6%, 95.7%, 96.9% |
|  | Quadratic | -23966.9 | -23902.1 | 475.4 | 17.1%, 14.4%, 45.1%, 17.9%, 5.5% | 97.5%, 89.3%, 90.6%, 77.9%, 92.8% |
|  | Cubic | -23614.7 | -23533.7 | 696.3 | 17.1%, 17.0%, 43.7%, 16.1%, 6.2% | 97.9%, 90.1%, 90.8%, 90.1%, 89.4% |
|  | Quartic | -23558.0 | 23460.8 | 105.3 | 17.0%, 16.8%, 43.2%, 6.4%, 16.6% | 97.9%, 91.0%, 90.9%, 90.7%, 82.6% |
| **Men** |  |  |  |  |  |  |
|  | Linear | -10911.1 | -10867.7 | - | 17.4%, 33.2%, 3.5%, 43.5%, 2.8% | 97.4%, 95.1%, 92.0%, 93.4%, 97.1% |
|  | Quadratic | -10335.2 | -10277.4 | 1143.7 | 18.8%, 14.2%, 26.9%, 34.2%, 5.8% | 98.9%, 81.4%, 92.9%, 92.6%, 92.4% |
|  | Cubic | -10209.4 | -10137.3 | 243.3 | 18.8%, 34.2%, 27.1%, 5.4%, 14.5% | 99.0%, 93.6%, 91.2%, 94.5%, 85.7% |
|  | Quartic | -10152.3 | -10065.7 | 106.5 | 18.7%, 33.9%, 14.0%, 27.4%, 5.9% | 98.8%, 92.5%, 88.5%, 92.8%, 94.8% |

Polynomial structures of a five-group model, BIC= Bayesian information criterion, AIC= Akaike information criterion.

### Supplemental Table S4: Model selection step 3. Combinations between cubic and quartic polynomial structures

| **Model** | **structure** | **BIC** | **AIC** | **Bayes factor**  **2log_e_(B10)** | **Predicted proportion per class** | **Posterior probability of class membership** |
| --- | --- | --- | --- | --- | --- | --- |
| **Women** |  |  |  |  |  |  |
|  | 3 3 3 3 3 | -23614.7 | -23533.7 | - | 17.1%, 17.0%, 43.7%, 16.1%, 6.1% | 97.9%, 80.5%, 90.8%, 90.5%, 89.4% |
|  | 4 3 3 3 3^*^ | -23647.8 | -23563.6 | -67.9 | 5.4%, 17.1%, 43.9%, 17.7%, 16.0% | 91.1%, 98.2%, 91.1%, 81.4%, 90.2% |
|  | 4 4 3 3 3 | -23604.1 | -23516.6 | 85.8 | 17.1%, 15.7%, 43.6%, 17.6%, 6.0% | 98.3%, 90.5%, 90.8%, 81.9%, 90.9% |
|  | 4 4 4 3 3 | -23566.2 | -23475.5 | 74.1 | 17.0%, 17.2%, 16.6%, 43.2%, 6.1% | 98.1%, 82.8%, 90.8%, 90.9%, 91.7% |
|  | 4 4 4 4 3 | -23592.7 | -23498.7 | -54.4 | 17.3%, 16.1%, 43.5%, 6.3%, 17.0% | 98.1%, 91.1%, 90.2%, 90.2%, 82.6% |
|  | **4 4 4 4 4** | **-23558.0** | **-23460** | **67.7** | **17.0%, 16.8%, 43.2%, 6.4%, 16.6%** | **97.9%, 91.0%, 90.9%, 90.6%, 82.6%** |
| **Men** |  |  |  |  |  |  |
|  | 3 3 3 3 3^*^ | -10209.5 | -10137.3 | - | 18.7%, 34.0%, 25.2%, 5.2%, 16.9% | 99.0%, 93.6%, 91.2%, 94.5%, 85.7% |
|  | 4 3 3 3 3 | -10244.0 | -10168.9 | -70.4 | 4.4%, 18.7%, 16.9%, 25.8%, 34.1% | 90.5%, 99.0%, 85.5%, 90.9%, 93.8% |
|  | 4 4 3 3 3^*^ | -10202.2 | -10124.2 | 82.0 | 18.7%, 25.1%, 34.1%, 16.9%, 5.2% | 99.2%, 91.1%, 93.7%, 86.1%, 94.5% |
|  | 4 4 4 3 3 | -10171.7 | -10090.9 | 59.3 | 18.6%, 16.4%, 25.9%, 33.3%, 5.7% | 98.8%, 86.2%, 92.5%, 93.1%, 94.1% |
|  | 4 4 4 4 3^*^ | -10185.0 | -10101.3 | -28.2 | 18.7%, 16.8%, 25.7%, 5.2%, 33.6% | 99.0%, 86.7%, 92.0%, 94.7%, 93.3% |
|  | **4 4 4 4 4** | **-10152.3** | **-10065.7** | **63.8** | **18.6%, 33.0%, 16.6%, 26.0%, 5.8%** | **98.8%, 92.5%, 88.5%, 92.8%, 94.9%** |

Asterisk (*) = model failed to converge, BIC= Bayesian information criterion, AIC= Akaike information criterion.

### Supplemental Figure S1. Individual level patterns within each trajectory group of a random sample of 25% of participants

## Supplemental References

1 Nagin, D. S. & Odgers, C. L. in *Annu. Rev. Clin. Psychol.* Vol. 6 109-138 (2010).

2 Lennon, H. *et al.* Framework to construct and interpret latent class trajectory modelling. *BMJ Open* **8**, e020683, doi:10.1136/bmjopen-2017-020683 (2018).

3 Kass, R. E. & Raftery, A. E. Bayes Factors. *Journal of the American Statistical Association* **90**, 773-795, doi:10.1080/01621459.1995.10476572 (1995).

4 Jones, B. L. & Nagin, D. S. A Note on a Stata Plugin for Estimating Group-based Trajectory Models. *Sociological Methods & Research* **42**, 608-613, doi:10.1177/0049124113503141 (2013).

5 Erosheva, E. A., Matsueda, R. L. & Telesca, D. Breaking Bad: Two Decades of Life-Course Data Analysis in Criminology, Developmental Psychology, and Beyond. *Annual Review of Statistics and Its Application* **1**, 301-332, doi:10.1146/annurev-statistics-022513-115701 (2014).
